# P-33. Survival analysis in pediatric patients with sepsis and treatment with IgG enriched with IgM and IgA

**DOI:** 10.1093/ofid/ofaf695.262

**Published:** 2026-01-11

**Authors:** Diana Donneys, José Gómez, Mónica Gil, Arieth Vargas, Gastón Castillo, J U A N P ROJAS

**Affiliations:** Libre University, CALI, Valle del Cauca, Colombia; Universidad Libre Seccional Cali., CALI, Valle del Cauca, Colombia; Universidad Libre Seccional Cali., CALI, Valle del Cauca, Colombia; Universidad Libre Seccional Cali., CALI, Valle del Cauca, Colombia; Fundación Clínica Infantil Club Noel de Cali, Colombia. Universidad Libre seccional Cali, CALI, Valle del Cauca, Colombia; Valle University, Libre University, CALI, Valle del Cauca, Colombia

## Abstract

**Background:**

Sepsis remains one of the leading causes of morbidity and mortality in pediatric intensive care units. Intravenous immunoglobulin enriched with IgM and IgA has been proposed as an adjunctive therapy to modulate the immune response and improve survival outcomes; however, evidence in the pediatric population is still limited.

Objective: To describe the survival of pediatric patients with sepsis treated with IgM- and IgA-enriched intravenous immunoglobulin in a pediatric intensive care unit.

**Table 1.**
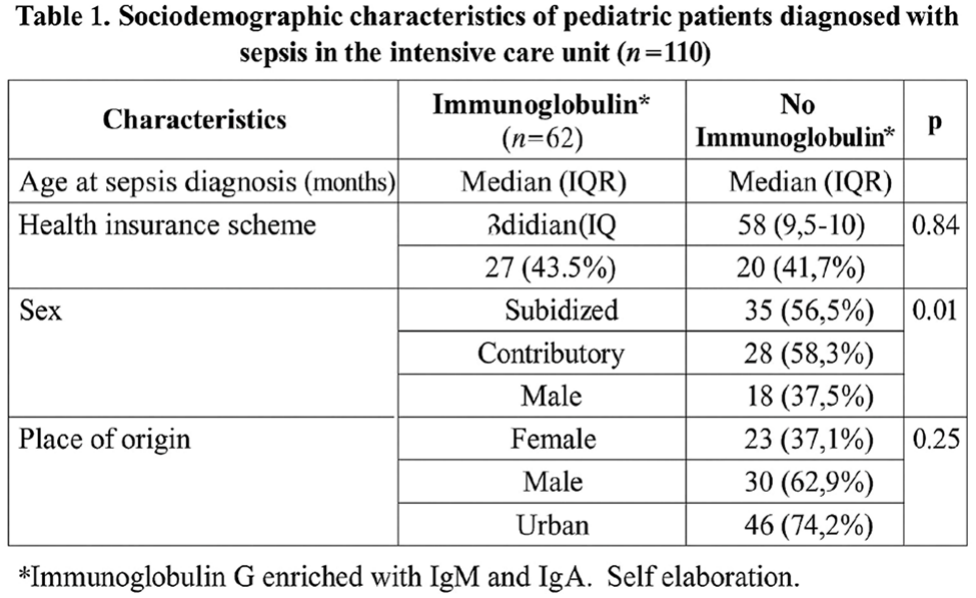
Sociodemographic characteristics of pediatric patients diagnosed with sepsis in the intensive care unit (n=110)

**Table 2.**
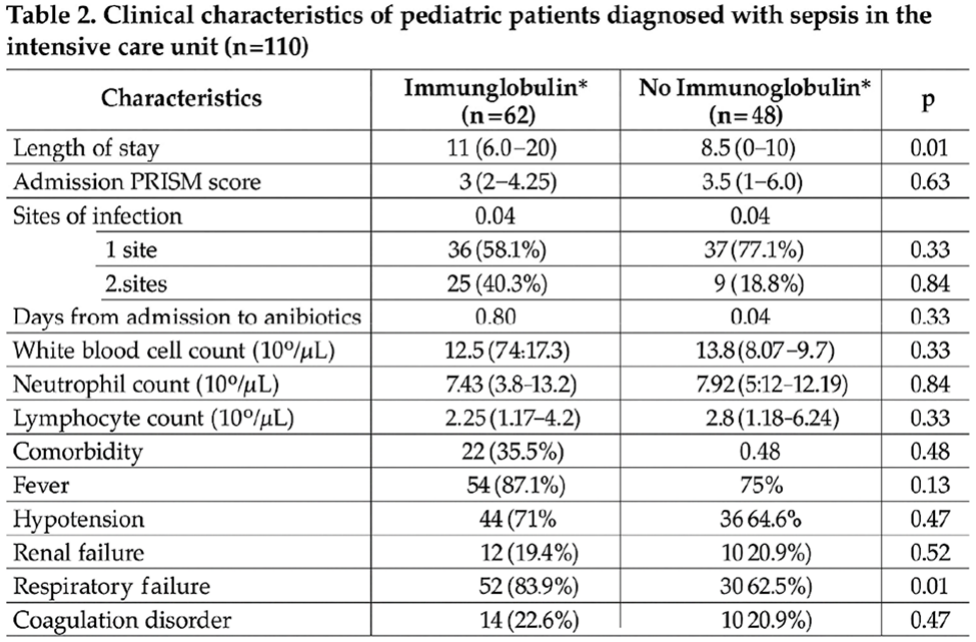
Characterization of humoral immunity in pediatric patients diagnosed with HTLV1 (n = 16)

**Methods:**

A retrospective cohort study was conducted between January 1, 2019, and December 31, 2023, in a pediatric intensive care unit. Patients aged 1 month to 18 years with a clinical diagnosis of sepsis—defined as severe infection with cardiovascular dysfunction (altered distal perfusion, hypotension, or use of vasoactive agents)—were included. Patients with inborn errors of immunity, prior use of subcutaneous or intravenous immunoglobulin, or secondary immunodeficiency were excluded. Survival was compared between patients who received IgM- and IgA-enriched intravenous immunoglobulin and those who did not. Kaplan-Meier analysis, Log-Rank test, and multivariate Cox regression were used.

**Figure 1.**
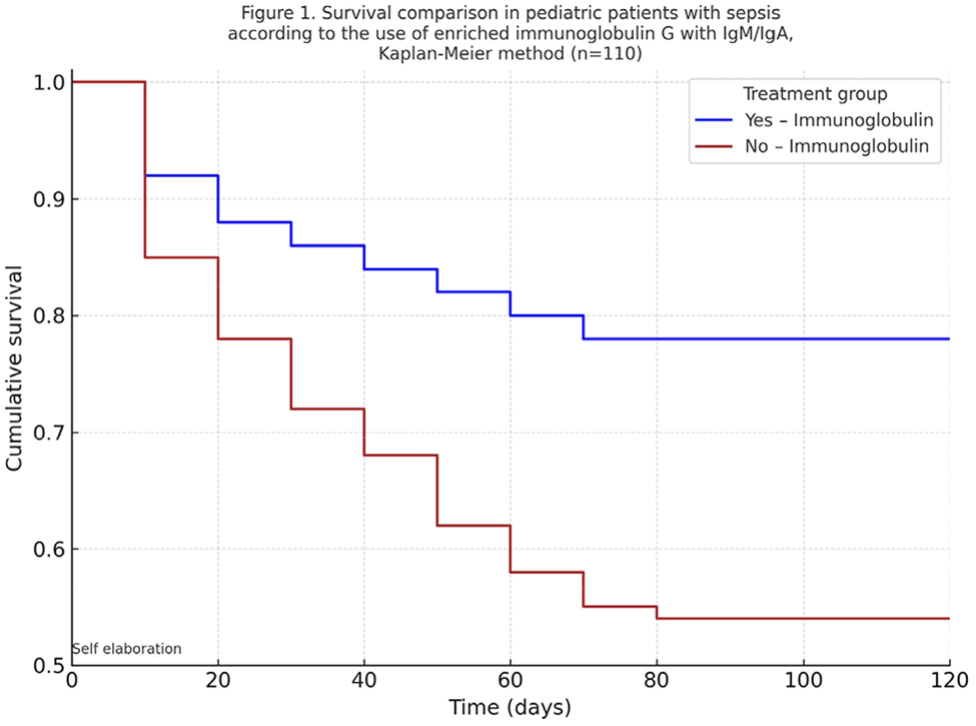
Survival comparison in pediatric patients with sepsis according to the use of Immunoglobulin G enriched with IgM/IgA, Kaplan-Meier method (n=110)

**Results:**

A total of 110 patients were included, of whom 62 received IgM- and IgA-enriched immunoglobulin. Mortality was lower in the treated group (12.9% vs. 29.2%; p = 0.03). Kaplan-Meier survival analysis showed a higher probability of survival in the immunoglobulin group (Log-Rank test, p = 0.03). In the multivariate analysis, immunoglobulin use was independently associated with a reduced risk of death (HR 0.30; 95% CI: 0.13–0.75; p = 0.01).

**Conclusion:**

The use of IgM- and IgA-enriched intravenous immunoglobulin was associated with improved survival in pediatric patients with sepsis, even among those with greater severity. These findings support its potential utility as an adjunctive therapy in pediatric sepsis.

**Disclosures:**

All Authors: No reported disclosures

